# On the Absorption of Insoluble Bodies

**Published:** 1849-01

**Authors:** 


*On the Absorption of Insoluble Bodies.* By M. MIALHE.—In our review of Mialhe's Traité de l'Art de Formuler (July Number, p. 37,) we mentioned that the experiments of Oesterlen, in which he found that charcoal, administered by the mouth to fowls and rabbits, passed into the blood, in which fluid the carbonaceous particles might be detected by the microscope, were opposed to Mialhe's doctrine, that "every substance, capable of exerting a remote action on the animal economy, is soluble, or susceptible of becoming so in the fluids of the body * * * such substances alone being capable of absorption."—(See also Journal for May, 1847, p. 488.) M. Mialhe has since repeated the experiments of Professor Oesterlen, and asserts that charcoal, when thus administered, does not pass into the circulation.—*Ret. of Med. Sci. from Gaz. Med. de Paris,* August 19.

